# *“if we don’t regroup, hunger will kill us…”:* a qualitative study on measures of physical distancing during covid-19 among internally displaced persons in Burkina Faso

**DOI:** 10.3389/fsoc.2023.1189235

**Published:** 2023-12-14

**Authors:** Kadidiatou Kadio, Antarou Ly, Adidjata Ouédraogo, Mohamed Ali Ag Ahmed, Sanni Yaya, Marie-Pierre Gagnon

**Affiliations:** ^1^Département Biomédical et de Santé Publique, Institut de recherche en sciences de la santé (IRSS), du Centre National de la Recherche scientifique et Technologique (CNRST), Ouagadougou, Burkina Faso; ^2^Fellow Pilote African Postdoctrorat Academy – PAPA, Goethe University Frankfurt, Frankfurt, Hesse, Germany; ^3^Department of Social and Preventive Medicine, Faculty of Medicine, Laval University, Quebec, QC, Canada; ^4^Centre de recherche du CHU de Québec, Laval University, Quebec, QC, Canada; ^5^Institute of Tropical Medicine Antwerp, Antwerp, Belgium; ^6^Faculté des sciences sociales, University of Ottawa, Ottawa, ON, Canada; ^7^Faculty of Nursing, Laval University, Quebec, QC, Canada

**Keywords:** internally displaced persons, COVID - 19, social distancing, physical distancing, Burkina Faso, Africa, sub-Saharan Africa, displaced people

## Abstract

This study contributes to the body of knowledge on IDPs in the context of security crisis related to terrorism. Very little research has been done on covid-19 amongst IDPs in Africa and this is one of the first studies in Burkina Faso. Our diversified sample allowed us to consider the discourses of humanitarian actors working with IDPs, but also the discourses of IDPs in a context of aggravated health and security crisis. The challenges encountered by IDPs in implementing physical distancing and the coping strategies have been documented. It showed some possible solutions that decision-makers could use in order to facilitate the appropriation of this measure by IDPs. This is a contribution to the field of applied human and social science research They will help to anticipate solutions in the event of a resurgence of covid-19 cases. In the current context, where the spread of the disease seems to be under control, concerted action should now be taken in the event of the detection of a case of covid-19 in the various IDP sites.

## 1 Background

The differential impacts of the covid-19 pandemic continues to be felt across countries and social groups (Doignon and Guilmoto, 2021; Bundervoet et al., 2022). People who are poor, mobile and displaced are disproportionately affected (Kobiané et al., 2020). On 9 March 2020, Burkina Faso announced its first cases of covid-19 (Ministère de la Santé, 2020). This pandemic appeared in a context of political and security crisis, characterised by the occupation of part of the territory by armed groups and the progressive increase in killings and crimes of all kinds (Hagberg et al., 2019; Dembele et al., 2020). Burkina Faso is estimated to be the second most affected country by terrorist attacks in Africa (Benedikter and Ouedraogo, 2019). This situation has had a negative impact on the living conditions of the population and has caused numerous internal displacements of people (OCHA, 2019; United Nations Office for the Coordination of United Nations Office for the Coordination of Humanitarian Affairs, 2020). As of April 302,022, Burkina Faso had just over 1,900,000 internally displaced persons (IDPs) (Sécretariat Permanent du CONASUR, 2023).

IDPs remain citizens of their country but live within host communities. Host communities provide accommodation for internally displaced families, or offer camps or sites intended for displaced persons to live in (UNHCR, 2007). The state is therefore legally responsible for the protection and well-being of IDPs. Forced displacement constitutes a tragic event for people (Dialma, 2002). They are weakened and more likely to suffer injustice and social and health inequalities (Cantor et al., 2021; Soma, 2021). « IDPs relocate in a different zone within their country, often in periphery of large urban centres, which constitutes the host community. In contrast to the host community, IDPs are more vulnerable to covid-19 because they usually lack access to decent housing (informal, unhealthy, cramped), adequate support and information to promote healthy living (Orendain and Djalante, 2021). Indeed, IDPs are often forced to settle in densely populated areas. They often have limited or no access to basic services (health facilities, sanitation, drinking water, etc.), thereby excluding them from most forms of aid and assistance (Alawa et al., 2020). These deficiencies disrupt their socio-economic stability and make them vulnerable to health problems such as covid-19 (Olanrewaju et al., 2018; Alawa et al., 2020). In most cases, health systems are unable to manage severe and critical forms of diseases such as covid-19 (Boum et al., 2021). Internal displacement thus appears to be one of the most damaging human mobility problems and urban phenomena, both for the people affected and for the host community (Orendain and Djalante, 2021). Yet it remains largely unaddressed in international discourse, advocacy, and research (UNOCHA, 2018).

In its quest to protect IDPs, the government of Burkina Faso has taken certain public health measures to curb the transmission of covid-19 and limit its spread (SIG, 2020). These include, for example, the prohibition of gatherings, and the closure of markets and places of worship (Bonnet et al., 2021). These so-called physical distancing measures consist of keeping a minimum distance of one metre between two people (Rocher and White, 2021; Sørensen et al., 2021). According to the World Health Organisation (WHO, 2020a) physical distancing is a response measure that consists of maintaining a safe distance or space between one person and another, especially if one person is coughing, sneezing or has a fever. Measures to promote physical distancing have been adopted in the covid-19 response plan in Burkina Faso. These included the use of non-contact greetings, maintaining a distance of at least one metre between individuals, staying at home, closing schools, work sites and places of worship, placing people in community quarantine, cancelling mass gatherings of more than 50 people, prohibiting the use of public transport, suspending non-essential services, and restricting travel.

All these measures were intended to break the chain of transmission of the disease. However, public health measures such as physical distancing to limit transmission of the virus that causes disease can be difficult to implement (Dubey et al., 2022). Research has shown that adopting the same approach across regions without taking context into account could reduce the effectiveness of these measures and lead to unintended negative consequences, such as loss of livelihoods and food insecurity (Chamberlain et al., 2022). In most of Africa’s cities, markets are centres of commercial exchange where opportunities to respect physical distances are limited (Rouamba et al., 2022). Informal urban settlements are developed, often overcrowded, with little spacing between housing units (Dubey et al., 2022). As a result, living spaces and essential social and hygienic facilities such as water sources and toilets are shared between households (Nyashanu et al., 2020; Ebekozien et al., 2022). In these contexts, physical and socioeconomic factors make it difficult, if not impossible, for individuals to effectively practise physical distancing (Wilkinson et al., 2020; Yap et al., 2020; Wamoyi et al., 2021) Maintaining physical distancing measures, especially when these measures have a significant impact on social norms, the economy and the psychological well-being of the population is challenging and more likely to limit survival in informal settlements and low-income populations (Wilkinson et al., 2020; Li et al., 2023).

However, their implementation was not without difficulty for the general population, it poses greater challenges for IDPs (Ozer et al., 2022). Indeed, for IDPs, these government measures remain difficult to implement because of their cramped and generally unhealthy spontaneous housing conditions (Orendain and Djalante, 2021). In Burkina Faso, the difficult living conditions and financial precariousness of IDPs as well as the lack of material and infrastructural resources for care exacerbate their vulnerability to covid-19 (Pam and Ahoure, 2021; Ozer et al., 2022). A study of IDPs in Mali found that crowded living conditions, beliefs and values, lack of toilets and drinking water at the sites, lack of financial resources and social pressure from religious leaders made it difficult to implement physical distancing measures (Ag Ahmed et al., 2021). Also, fear of stigmatisation, low literacy levels and language barriers are cited as difficulties in changing the behaviour of IDPs towards covid-19 (Ly et al., 2022).

Although Covid 19 appears to be over, the present research is still relevant. Recently, a new variant of the virus entered circulation (WHO, 2023). The issue of IDPs in Burkina Faso is a topical one, and their numbers are increasing because of insecurity. Yet little research has been carried out on this category of the population, whose living conditions expose them to greater health risks. Studies on Covid 19 have most often focused on the economic impact, neglecting to analyze the realities experienced by vulnerable populations such as IDPs because of the imposition of public health measures such as physical distancing (Li et al., 2023). For this reason, it is important to better understand their situations, so that public health measures can consider their specific constraints and social dynamics, in order to plan and deploy future interventions. Thus, the aim of this study aimed to to document the challenges faced by humanitarian actors and IDPs in the application of physical distancing measures in the commune of Kaya. The objectives were to: (1) explore knowledge regarding the application of physical distancing measures amongst IDPs and associated challenges, and (2) describe the strategies used by IDPs to overcome the difficulties encountered in the application of these measures.

## 2 Methods

### 2.1 Research setting

The study was conducted in Burkina Faso, in the commune of Kaya, province of Sanmatenga and the capital of the centre-north region (ONTB, 2023). The region hosts the largest number of IDPs. As of 28 February 2022, the number of IDPs in the region was estimated at 652,159 out of a national total of 1,814,283 (Sécretariat Permanent du CONASUR, 2023). The province of Sanmatenga was home to approximately 57% of IDPs. In addition, the commune of Kaya had the largest number of IDPs during the same period, i.e., 123,610 (Ozer et al., 2022). The majority of IDPs are children (62.7%), women (22.9%) and men (14.4%). Their care is coordinated by the decentralised services of the Ministry of Humanitarian Action with the support of several humanitarian actors. In Kaya, IDPs are either hosted by households or live in accommodation sites.^1^

### 2.2 Identification of participating sites

The regional directorate for gender, national solidarity, family and humanitarian action in the centre-north was the entry point for this study. It helped us to understand the organisation of the IDP reception sites and directed us to the provincial directorate of social action, which facilitated contact with the IDP site managers. Site managers are delocalized social action agents, usually working in pairs within each site. In the city of Kaya, the IDPs are installed by affinity according to their locality of origin and by wave of arrival. They are grouped together in 13 sites located mainly in the outskirts of the town, particularly in undeveloped areas or spontaneous settlements. These agents are supported by a committee of ten (10) people. This committee is composed of representatives of the IDPs and the host community. All activities carried out in the sites are organised with the support of this local committee.

Study participants were recruited from two IDP sites were used for the recruitment of study participants. These sites were chosen according to the type of housing provided to the IDPs (housing built by the UNHCR, and housing built by the IDPs). Considering ongoing activities (research or intervention) in the sites, the research assistants were put in contact with the management committee of each site to recruit participants. There were four research assistants, each trained in sociology. The social workers ensured that no other activities from different structures were going on at the same site at the same time. The idea was not to overload the IDPs or the people working on securing the site.

### 2.3 Description of study sites

In choosing our study sites, we wanted them to have different profiles in order to diversify our data sources by taking into account certain criteria (type of habitat, population and organisation). Thus, at site 1, IDPs are mostly housed in rented houses or even houses that they built themselves on portions of land granted by the indigenous population. The IDPs are scattered and often confused with the indigenous population. Some IDPs even bought land to build on. This site has been housing IDPs for over 3 years. It is managed by two agents of the social action services.

In contrast, at site 2, IDPs are housed mostly in tents built by the UNHCR. This site is smaller in size and has been housing IDPs for 1 year. Compared to site 1, site 2 is more overcrowded. The site is fenced, but the fence is disappearing due to the high number of IDPs. Some of the IDPs are located outside the boundaries of the fence. Site 1 is mainly inhabited by Mossi and Foulsés, whilst on site 2, in addition to the Mossi and Foulsés, there are also Fulani. Several humanitarian NGOs are involved on both sites.

### 2.4 Participant recruitment

Two categories of respondents participated in the research: the IDPs and the actors involved in the care of IDPs, namely humanitarian actors and social and health authorities.

Two research assistants per site were put in touch with each site’s management committee to recruit respondents. Residents of the sites were informed by the interim of their managers inviting them to participate voluntarily in the research. At each site, IDPs were identified in order of arrival at the location where the interviews were to be held. Other criteria such as age, gender, marital status and origin were considered for the selection of participants. The actors involved in the care of IDPs were selected according to their availability and involvement. Humanitarian actors were selected from amongst the agents and managers of NGOs and local associations that supported IDPs (n = 8). The sociosanitary and administrative authorities were chosen from amongst the agents of the provincial directorate of social action, health agents who worked in the care of IDPs at the sites, community health agents, and staff of the Kaya health district (n = 10). The total sample consisted of 29 IDPs and 18 humanitarian actors, 26 of whom were men and 21 women. Twenty of them had no education. The average age of the IDPs was estimated at 38.4 years, the lowest age was 20 years and the highest was 63 years.

### 2.5 Data collection

On the day of the interview, each participant was informed about the elements of the research (the form of collection, the potential risks and benefits of participation, data confidentiality and the right to withdraw) and their signed and verbal consent was received. We conducted a total of 47 semi-structured face-to-face interviews that were audiorecorded with the consent of the interviewees. Two flexible interview guides were developed, one for humanitarian actors and sociosanitary authorities, and the other for IDPs. The themes of the interviews were: (1) knowledge of the different actors (humanitarian actors and IDPs) about covid-19 and physical distancing measures; (2) difficulties and challenges encountered by IDPs and humanitarian actors; and (3) the adjustments made by IDPs and humanitarian actors in applying physical distancing measures. Participants were encouraged to share their experiences and perceptions of physical distancing. Open-ended questions were preferred to encourage rich, detailed, and nuanced responses, but also to allow new avenues to be explored during the interviews. In fact, the guide included incentives to delve deeper into specific aspects, whilst allowing participants to raise issues they felt were important. The principle of triangulation was applied to increase the internal validity of our results. This consisted of diversifying the participants in order to cross-checque information (Olivier de Sardan, 2008) to identify convergences or divergences. The saturation principle (^1004,1003^) allowed us to end the field survey, i.e., the data collected no longer provided new information. Data collection took place between June 21–30, 2022, and interviews were conducted by four research assistants trained in sociology.

### 2.6 Data analysis

A descriptive qualitative study was conducted, which allows understanding a complex and detailed phenomenon from the meanings that people who experience it give to it (Creswell, 2013). The data analysis was carried out in several stages using the thematic discourse analysis (Paillé and Mucchielli, 2012). First, the interviews were transcribed in full. After a rereading of the speeches, the interviews were imported into the NVivo 12 software for the organisation of the corpus. We designed a non-rigid coding grid from the preliminary reading and the interview grids to consider emerging themes. Based on this preliminary grid, each interview was segmented and coded by unit of meaning or category of response in nodes (free and hierarchical). Careful reading of the content of each node and sub-node allowed to deepen the analysis by creating or merging the nodes. This allowed for an in-depth study of the discourse segments with the aim of transforming them into explicit themes.

### 2.7 Ethics and confidentiality

The study was approved on 12 August 2020 by the Ethics and Health Research Committee of Burkina Faso under number 2020–0-152. Informed consent was obtained from each participant after explaining the objectives of the study and the risks involved. Particular care was taken to respect the norms of confidentiality and non-disclosure of the participants’ identities. In reporting participants’ respsones, identifying information has been removed and participants have been assigned psudonyms based on type of participant, gender and project site. Project staff signed a confidentiality and good ethical practise agreement. During data collection, hand hygiene, barrier gestures and face covering were observed by the research assistants.

## 3 Results

### 3.1 IDPs’ knowledge of covid-19 and physical distancing measures

Some IDPs have been informed about the disease through radio, TV, local associations and informal talks,. Also, humanitarian organisations and associations have conducted several awareness-raising activities that have raised awareness amongst IDPs about the issues surrounding the pandemic. For the majority of IDPs, covid-19 is a dangerous disease because it attacks the respiratory tract and is sometimes difficult to manage. They also maintain that it is a disease that has claimed many victims in the world, hence the need to protect themselves.

*"If you get this disease, you can't breathe, whereas the root of man is breathing. If you come to say that someone is no more, his breath is gone. If the disease wants to get you, it attacks the breath. With other patients, despite respiratory assistance, it is not possible to treat them, the lungs are affected. It is a really dangerous disease*" (IDP-site1-female)

Awareness-raising sessions on covid-19 by humanitarian actors were very often accompanied by the donation of hand-washing kits, hydroalcoholic gels or disinfectants and protective masks or muffs. They raised awareness amongst IDPs about the existence of the disease, but also about how to avoid becoming infected with covid-19. Although there were no cases of covid-19 in the IDP sites before and during our study, the commune of Kaya had recorded positive cases of covid-19 (n = 126). This led to the adoption of response measures, including physical distancing. For the IDPs, physical distancing was defined as the prohibition of gatherings, the closure of public places (places of prayer, markets, etc.), the distance of one metre between people, the prohibition of shaking hands, and the limitation of visits to third parties. For them, these different measures make it possible to limit and avoid contamination by the disease. They explain that the one-metre distance between people avoids exposure and contracting the disease through respiratory droplets and aerosols when an infected person breathes, coughs, speaks or sneezes.

*"It means that if you put a metre between you, even if one of you sneezed your friend won't get the disease. If you coughed, your spit won't reach another person. And if you have a cold and you took a handkerchief to blow your nose, you should not put it anywhere else, you should put it in the dustbin*" (IDP-site2-female)

For IDPs, physical distancing is therefore an effective measure to avoid exposure to the disease when meeting other people. The same is true for prohibiting gatherings and closing public places. They explained that access to public spaces can favour the spread of the disease insofar as people who meet are likely to be contact cases. Hence the relevance of respecting these measures in order to contain the spread of the disease. One of the IDPs explained that:

*"This is also a measure because if people gather in large numbers that's where the disease can spread from”* (IDP-site1-male)

Even though IDPs were aware of the dangers of the disease and had knowledge of the response measures, particularly physical distancing, the implementation of this measure was often a dilemma for them due to their social, economic and contextual realities.

### 3.2 Challenges faced by the authorities and/or humanitarian actors

One of the challenges for humanitarian actors was communication. The aim of this communication is to make IDPs aware of the risks posed by covid-19. At the time of data collection in June 2021, we found that the issue of the pandemic was relegated to the background. During the first twelve (12) months of the pandemic, all actors were more open to complying with the response measures, but as time went by, the pandemic gradually took a back seat. In such situations, humanitarian actors were faced with the challenges of communication, i.e., conveying the right information to convince IDPs to adhere to protective measures. One humanitarian actor explained that:

*"Don't go out, don't come in, containment is always the same word. We have to make sure that people understand, accept and follow the instructions. If they understand, they can follow because if they are informed. But if they don't have any knowledge, they can't follow the instructions. If they know all the information about the disease, they can follow the instructions and people will accept to follow the barrier measures. Apart from that, there will be no difficulty, people will be able to respect the barrier measures …* " (OH-volunteer, former ASBC)

Another challenge faced by humanitarian actors was reconciling physical distancing measures with the social values of IDPs. Some of them pointed out that it was almost impossible for them to enforce certain physical distancing measures in the residence sites. This is explained by the communal way of life of IDPs. They explained that it is only in health centres and humanitarian structures that the one metre distance between people can be enforced. One of them argues that:

*"Because in community life it doesn't even work. From the moment that in the family we get together even to eat, it will be difficult… We gather around a dish to eat and you say, 'Do 1 m, 1 m, 1 m, how do we do it? So social distancing in community is not going to work”* (SI- health worker, 26 June 2021)

In order to meet these challenges and get IDPs to respect public health measures, including physical distancing, administrative and humanitarian actors believe that IDP community leaders should be involved. They believe that if measures are imposed, IDPs will apply them as a courtesy to their presence, but will not respect them once the humanitarian actors have left. Hence the need for them to involve their community leaders. The latter should act as relays in the sites to discuss with IDPs the importance of respecting these measures individually and collectively.

### 3.3 Difficulties encountered by IDPs in implementing physical distancing measures

The implementation of these physical distancing measures by IDPs entails many difficulties, most of which are socio-economic.

On the socio-cultural level, the vast majority of IDPs and humanitarian actors mentioned the incompatibility of physical distance with their social values. For them, the presence of relatives at social events is important in maintaining solidarity links. Forced displacement also strengthened solidarity amongst IDPs. The latter settled in the reception site with a logic of grouping by area of origin. Thus, they tried to reconstitute the villages of origin, in order to maintain mutual aid and moral support, given that they share common painful experiences. In such a context, asking them not to attend social events because of the need for physical distancing is for IDPs a renunciation of fundamental human values. Similarly, the observation of a metre of distance between people is seen as an impediment to having deep discussions. They explained that one cannot confide in or listen to a person whilst respecting physical distance. One of the interviewees argued that:

*"Distancing oneself is difficult, humanism means sitting next to the other. Wallaye, that can lead to problems because when we know each other and we don't go to our respective ceremonies that can also lead to a problem. If we say, not to get closer, that can also be like a suffering*" (IDP-site1-female).

In order to support their families, the IDPs, who are mostly women, engage in various income-generating activities such as selling sand or petty trade. They also work as household help in families and in restaurants. With the implementation of measures to promote physical distancing, households and restaurants have become inaccessible to IDPs. This has resulted in a loss of income for IDPs who struggled to cover some of the basic needs of their households

*"Ahi this has caused a lot of problems, we who are the watba (coming or IDPs). We go out to have clothes to wash to come and cook for our children and husbands. If they come and tell us not to go out, we will only die (laughs). If we don't get a job to work, to be able to feed our families, that's not death? um, that's also a problem*" (IDP-site2-female)

For physical distancing, IDPs mentioned the impossibility of applying this measure in their context because of the communal, social nature in which they live. One of the IDPs interviewed supported this argument:

*"Because communally it doesn't even work, because we get together to eat, we don't eat with our own plate like over there (laughs). We gather around a plate to eat and you say, do 1 m, 1 m, 1 m, how do we do it? So physical communal distancing is not going to work”* (IDP-site-1male)

### 3.4 Strategies put in place to adjust to physical distancing measures

For IDPs, it is difficult to find adjustments in the application of physical distancing. In order to cope with their precariousness, they have two alternatives: (i) they rely on humanitarian aid or (ii) on the income they obtain from income-generating activities. The conditions under which IDPs are cared for and their living conditions make them particularly vulnerable to the application of measures that promote physical distancing. They explain that they are obliged to gather on the premises of social services or humanitarian actors. For instance, some humanitarian actors organised food distribution activities in groups of 50 IDPs in order to respect the minimum distance of 1 metre between them. Moreover, the food donations they receive cannot cover their needs over a long period of time. Indeed, although some of them receive monthly assistance, many are forced to make do with one-off donations. As a result, they go around every day to the structures likely to provide them with food kits. Thus, even when they are informed that they will not receive food kits, IDPs gather at the various social services in the hope of receiving aid. We observed this during data collection. One of them said that she could not find any adjustment measures in this kind of situation where she had to choose between protecting herself from the disease or fetching the daily food for the family. So, as this IDP explains, this is a choice between physical distancing and survival:

*"Often, we take a bag of millet and we share it with four people and often we share it with six people. When six people have to share one bag of millet, there is nothing in it. It won't even be enough for two days' food, so you have to leave the next day, maybe you'll still have some”* (IDP-site1-female)

The same applies to access to income-generating opportunities. The closure of public places (bars, restaurants, markets, etc.) and the reluctance of households to receive strangers are factors that forced IDPs to adopt certain measures that promote physical distancing. They did so because they had to, not because they wanted to protect themselves from the disease.

Intervieweed IDPs did not mention that they used any adjustment strategy to comply with physical distanciation measures. Some IDPs felt that awareness raising should continue, others felt that there was no adjustment possible for this measure, as they felt that they live in a community and are called upon to come together at any time to receive help. This IDP from site 1 added that:

*"If we don't get together, hunger will kill us. If we don't go to social action to get something for a little bit, hunger will kill us. There is no adjustment that is possible”*

Thus, the implementation of physical distancing would increase the vulnerability of IDPs. On the one hand, they were very limited in their access to income-generating activities. On the other hand, access to humanitarian services was also limited.

## 4 Discussion

The aim of this study was to identify the knowledge and challenges faced by authorities and/or humanitarian actors in applying physical distancing to IDPs, and to explore the difficulties encountered by IDPs in applying this public health measure, as well as the adjustments made by the different actors to overcome the difficulties encountered.

The results showed that the common challenge for the administrative authorities and humanitarian actors was that of communication, i.e., getting the “real information” to IDPs so that they can appropriate it. This communication challenge was unanimously recognised by the humanitarian actors to reduce the false information on covid-19 that was circulating amongst IDPs. It was important for IDPs to have access to accurate information about the disease, as misinformation about physical distancing measures could reinforce social isolation and harm their well-being (Mesa Vieira et al., 2020). A study amongst IDPs in Sudan showed the presence of misinformation about covid-19: belived that spraying the body with alcohol or chlorine helps eliminate the virus mosquito bites could transmit the disease; that spraying the body with alcohol or chlorine helps eliminate the virus, that regular washing with saline solution helps prevent infection, that eating garlic helps prevent the disease. The study explains that misinformation occurred because most respondents (IDPs) obtained information about covid-19 from their relatives and friends. They did not have access to other sources of information such as television, radio, social media and other internet services in conflict areas (Abdelmalik et al., 2022).

Another major challenge identified by authorities and/or humanitarian actors was the acceptability of IDPs to apply physical distancing. The IDPs themselves acknowledge having faced difficulties in applying physical distancing. The context of humanitarian emergencies complicates the task of actors during social activities. In some cases, physical distancing is difficult or even impossible during the distribution of emergency and food kits because of the large number of IDPs at the reception of food distribution sites. These high numbers could be the consequence of organisational dysfunction and weakness in the coordination of activities that support the care of IDPs. In addition, some IDPs and aid workers argue that the communal way of life and the traumatic displacement trajectory exacerbated by terrorist violence mean that the adoption and application of measures that promote physical distancing are often perceived as social isolation or even abandonment. The psychological consequences of physical distancing due to the fact that it reinforces social isolation has also been mentioned by other research (Song and Ventevogel, 2020; WHO, 2020b; Song, 2021).

Inadequate housing and cramped premises make the application of physical distancing in IDP households and sites virtually impossible. The application of physical distancing had also been recognised as a major difficulty amongst IDPs in Mali due to cramped premises and inadequate housing infrastructure (Ag Ahmed et al., 2021). A study in Congo mentioned the inability to practise physical distancing due to overcrowded conditions in the reception sites, in particular the fact that several families share a dormitory. Although 89% of the participants in this study mentioned that avoiding physical contact is a measure to prevent covid-19, 70% had been in close contact with a family member or an outsider (Claude et al., 2020). Other studies have recognised the impossibility of physical distancing in IDP camps, as many people are in close contact and gather in large groups (Kluge et al., 2020; Vince, 2020).

In response to these challenges and difficulties, humanitarian actors had adopted adjustment measures such as reducing the number of people per group during food distribution. In contrast, IDPs did not find it possible to adjust to the physical distancing in their context of cramped premises and insufficient housing infrastructure. For this reason, humanitarian actors proposed to reinforce certain public health measures such as hand washing and the wearing of masks to curb any possibility of spreading the virus in case of contamination.

Furthermore, as it has already been done for prisons, the administrative and humanitarian authorities can take steps to relieve the overcrowding in IDP camps by creating new ones. This will allow for better control of the number of people in the camps and therefore better enforcement of social distancing measures. Similar arrangements have been made in countries such as Nigeria, Sudan and South Sudan (OIM, 2020).

This study contributes to the body of knowledge on IDPs in the context of security crisis related to terrorism. Very little research has been done on covid-19 amongst IDPs in Africa and this is one of the first studies in Burkina Faso. Our diversified sample allowed us to consider the discourses of humanitarian actors working with IDPs, but also the discourses of IDPs in a context of aggravated health and security crisis. The diversity of respondents from both humanitarian actors and IDPs allowed us to triangulate our sources of information, thereby improving the robustness of the findings. The aforementioned strengths notwithstanding, this study has some limitations. It does not claim to be representative of all IDPs in Burkina Faso. Although we have reached information saturation, we recognise that the results are not generalisable and that future research raising similar issues may validate or contradict our findings due to nuances in contexts. Furthermore, the study was conducted in June 2021 when there were no reported cases of covid-19 in the IDP population studied. This did not allow us to capture the respondents’ experience of physical distancing in the context of awareness of positive cases in their environment.

## 5 Conclusion

This study documented the challenges faced by humanitarian actors, but also identified the difficulties faced by IDPs in implementing covid-19 physical distancing measures. Although the study was carried out in June 2021, more than a year after the first cases of covid-19 were reported in Burkina Faso, the results obtained can impact on decision-making and alleviate the difficulties faced by IDPs. They will help to anticipate solutions in the event of a resurgence of covid-19 cases. It showed some possible solutions that decision-makers could use in order to facilitate the appropriation of this measure by IDPs. In the current context, where the spread of the disease seems to be under control, concerted action should now be taken in the event of the detection of a case of covid-19 in the various IDP sites.

## Data availability statement

The datasets presented in this article are not readily available because full transcripts cannot be shared publicly due to potentially identifying information. The content and words of respondents in interviews could potentially be used to identify individuals. Even anonymisation could pose risks to confidentiality. Data are available upon request to the Comité d’éthique pour la recherche en santé in Burkina Faso (+226 72757187). Requests to access the datasets should be directed to the Comité d’éthique pour la recherche en santé in Burkina Faso (+226 72757187).

## Ethics statement

The studies involving humans were approved by Ethics and Health Research Committee of Burkina Faso. The studies were conducted in accordance with the local legislation and institutional requirements. Written informed consent for participation in this study was provided by the participants' legal guardians/next of kin.

## Author contributions

KK: Conceptualization, Data curation, Formal analysis, Methodology, writing original draft, writing review & editing. AL: Conceptualization, Methodology, Supervision Investigation, writing review & editing. AO: Investigation, Formal analysis, Writing original draft. MAAA: Conceptualization, Methodology, review & editing. SY: Conceptualization, Methodology, review & editing. M-PG: Conceptualization, Methodology, review & editing.

## Footnotes

^1^contacts_des_acteurs_humanitaires_centre-nord_05292020.pdf: Centre-Nord Region, Burkina Faso, 29 May 2020: https://www.humanitarianresponse.info/sites/www.humanitarianresponse.info/files/documents/files/contacts_des_acteurs_humanitaires_centre-nord_05292020.pdf,
